# A Machine Learning Method to Identify Umami Peptide Sequences by Using Multiplicative LSTM Embedded Features

**DOI:** 10.3390/foods12071498

**Published:** 2023-04-02

**Authors:** Jici Jiang, Jiayu Li, Junxian Li, Hongdi Pei, Mingxin Li, Quan Zou, Zhibin Lv

**Affiliations:** 1 College of Biomedical Engineering, Sichuan University, Chengdu 610065, China; 2 College of Life Science, Sichuan University, Chengdu 610065, China; 3 Wu Yuzhang Honors College, Sichuan University, Chengdu 610065, China; 4 Institute of Fundamental and Frontier Sciences, University of Electronic Science and Technology of China, Chengdu 610054, China; 5 Yangtze Delta Region Institute (Quzhou), University of Electronic Science and Technology of China, Quzhou 324000, China

**Keywords:** umami peptide, deep representation learning, SMOTE, ANOVA, light gradient boosting, mutual information, multiplicative LSTM

## Abstract

Umami peptides enhance the umami taste of food and have good food processing properties, nutritional value, and numerous potential applications. Wet testing for the identification of umami peptides is a time-consuming and expensive process. Here, we report the iUmami-DRLF that uses a logistic regression (LR) method solely based on the deep learning pre-trained neural network feature extraction method, unified representation (UniRep based on multiplicative LSTM), for feature extraction from the peptide sequences. The findings demonstrate that deep learning representation learning significantly enhanced the capability of models in identifying umami peptides and predictive precision solely based on peptide sequence information. The newly validated taste sequences were also used to test the iUmami-DRLF and other predictors, and the result indicates that the iUmami-DRLF has better robustness and accuracy and remains valid at higher probability thresholds. The iUmami-DRLF method can aid further studies on enhancing the umami flavor of food for satisfying the need for an umami-flavored diet.

## 1. Introduction

Umami taste has been widely accepted as the fifth basic taste, along with the four other basic tastes of sweet, sour, salty, and bitter [1]. Umami substances are important for enhancing the flavor of food and healthy eating [2]. Umami peptides frequently contain aspartic acid, glutamic acid, asparagine, or glutamine residues. Peptides that contain these umami amino acids may or may not have an umami flavor, and may instead have a bitter flavor in the absence of umami amino acids [3]. Umami peptides are a novel class of umami agents with numerous potential uses and a distinctive flavor. Additionally, these peptides act synergistically with other umami compounds to enhance the sweetness of sweet items and the saltiness of salty items, but reduce sour and bitter tastes, thus softening the taste. Umami is a very important factor affecting the quality of food, and increasing the content of umami substances in the food improves the overall palatability.

Wet tests are costly and time-consuming when identifying umami peptides. The post-genomic era’s proliferation of peptide sequence databases [4,5] has had a significant impact on the practical application of automated mathematical methods for the discovery of novel umami peptides. The development of umami peptide prediction tools using deep representation learning features has attracted increasing interest in the field of bioinformatics [6,7,8]. Umami-SCM [9] was developed in 2020 and uses the scoring card method (SCM). It is combined with the propensity score of amino acids and dipeptides for identifying umami peptides [10]. The independent test accuracy of this method was reported to be 0.865 and the predictor performed better in 10-fold cross-validation tests than in existing methods. Charoenkwan et al. developed UMPred-FRL in 2021 [11], which integrated seven different traditional feature codes for constructing the umami peptide classifier. Jiang et al. proposed iUP-BERT in 2022 [12], which is based on the use of a single deep representational learning feature encoding method (BERT: bidirectional encoder representations from transformer). Compared to Umami-SCM and UMPred-FRL, iUP-BERT has superior performance in both independent testing and cross-validation. Despite notable advancements in the field, particularly in terms of independent testing, machine learning (ML)-based umami peptide detection algorithms that rely exclusively on sequence data still need to significantly improve in terms of performance. The above predictors are still not accurate enough, and there is still room for improvement. For instance, we found that iUP-BERT was not as robust as expected.

Representation learning [13,14] comprises ML techniques that enable the automatic identification of representations from raw data for feature detection or classification. This eliminates the need for manual feature engineering and enables machines to learn the features of protein or peptide sequences, and apply them to perform specific tasks. During depth evaluation in representation learning, ML techniques are used for transforming data from the original representation to a new representation that preserves the information necessary for the object of interest [15,16,17,18,19,20,21,22], while discarding the redundant information [23,24,25,26,27,28,29,30,31]. Sequence-based deep representation learning has been recognized as an innovative and efficient construction in protein and peptide research for protein feature prediction [32,33,34,35,36,37,38,39], including the unified representation (UniRep) method [40] and BiLSTM [41]. 

**Figure 1:**
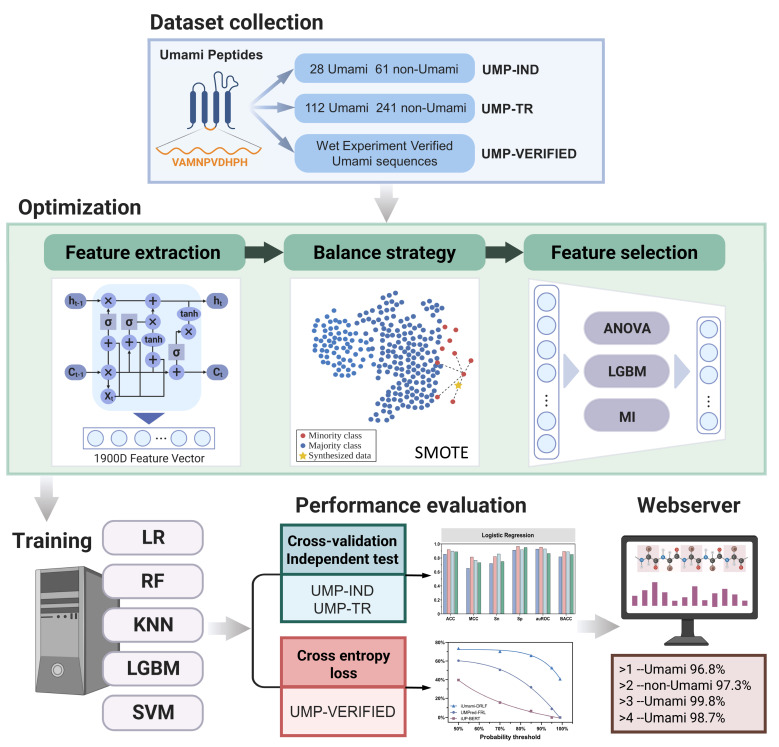
Overview of model development. The pre-trained UniRep sequence embedding model was used to embed the peptide sequences into eigenvectors. The peptide sequences were converted into 1900-dimensional (D) UniRep eigenvectors. The synthetic minority over-sampling technique (SMOTE) was used for balancing the imbalanced data. These features were used as inputs to the k-nearest neighbors (KNN), logistic regression (LR), support vector machine (SVM), random forest (RF), and light gradient boosting machine (LGBM) predictor algorithms. Feature extraction was performed for model optimization using analysis of variance (ANOVA), LGBM, and mutual information (MI). The selected feature sets were subjected to another round of analysis using the three feature extraction algorithms and various hyperparameters. The final optimized model was developed by comparison of model performance in 10-fold cross-validation and independent tests. Based on the 91 wet-test validated umami peptide sequences reported in the latest research (UMP-VERIFIED), we evaluated iUmami-DRLF in comparison to state-of-the-art methods.

In this study, sequence-based unified representation (UniRep) features based on multiplicative LSTM were solely used for developing an ML-based model, iUmami-DRLF, for the identification of umami peptides. iUmami-DRLF showed exceptional outcomes in the independent tests and 10-fold cross-validation studies. The obtained results had high accuracy, and more importantly, the results of independent testing proved iUmami-DRLF to be far superior to the current techniques and conventional non-deep representation learning techniques. Additionally, iUmami-DRLF has a wider range of applications and excellent umami peptide discrimination potential. The iUmami-DRLF predictor outperformed the conventional forecasting techniques in the 10-fold cross-validation tests (Sn = 0.959 and auROC = 0.957) and independent tests (ACC = 0.921, MCC = 0.815, Sn = 0.821, Sp = 0.967, auROC = 0.956, and BACC = 0.894). The independent test accuracy of iUmami-DRLF is improved by 2.45% as compared with that of iUP-BERT. The effects of various feature analysis methods and various deep representation learning features for classification results were examined using the unified manifold approximation and projection (UMAP) dimensionality reduction approach. Compared with other SOTA methods, iUmami-DRLF in this study has higher accuracy under various probability thresholds, and shows better robustness and generalization performance. The steps performed for the construction of iUmami-DRLF are depicted in Figure 1.

## 2. Materials and Methods

### 2.1. Benchmark Dataset

In this work, the model was developed using the updated benchmark dataset from iUmami-SCM [9], which also facilitates future comparisons. The BIOPEP-UWM [4] database and experimentally verified umami peptides were included in the positive dataset, while bitter non-umami peptides were included in the negative dataset. The UMP442 benchmark dataset, which contains 304 non-umami peptides and 140 umami peptides, is acquired after data cleaning. To avoid the prediction model becoming overfit, the dataset was arbitrarily split into a training subset UMP-TR, and an independent test peptide subset denoted as UMP-IND. The UMP-TR dataset comprised 112 umami and 241 non-umami peptides, while the UMP-IND dataset comprised 28 umami and 61 non-umami peptides. The URL for both datasets is http://public.aibiochem.net/peptides/iUmami-DRLF/ (accessed on 1 April 2023). To validate the accuracy and robustness of our model, we also collected 91 wet-experiment verified umami peptide sequences from the latest research (please see Supplementary Table S2). The 91 wet-experiment verified umami peptides dataset was named UMP-VERIFIED.

### 2.2. Feature Extraction

For UniRep [40], a total of 24 million core amino acid sequences from UniRef50 were used for training the UniRep model. By identifying the subsequent amino acid by reducing cross-entropy losses, the model learns how to accurately express proteins after training. Using the trained model, the input sequence was represented as a single fixed-length vector (hidden state) with a multiplicative long short-term memory (mLSTM) encoder. The ideal ML model was trained using the output vector representation. Supervised learning is achieved in various bioinformatics tasks by using the input sequence as a personality.

First, a matrix containing the sequences of S amino acid residues was integrated using the single thermal code $R^{S\times 10}$. The matrix was then put through into the mLSTM encoder to generate an output hidden state of $R^{1900\times S}$ as an embedding matrix. The 1900-dimensional (D) UniRep feature vector was finally derived using an average pooling operation. 

The equations used by the mLSTM encoder for performing the calculations are provided hereafter (Equations (1)–(7)). Where $m_{t}\;$represents the current intermediate multiplication state, ${\hat{h}}_{t}$ is the input before the hidden state, $f_{t}$ represents the forgotten gate, $i_{t}$ represents the input gate, $o_{t}$ stands the output gate, $h_{t}$ stands the hidden state for output, and $C_{t}$ is the current unit state.

(1) $$m_{t}=\left(X_{t}W_{xm}\right)⨂\left(W_{hm}h_{t-1}\right)$$

(2) $${\hat{h}}_{t}=\left(W_{mh}m_{t}+W_{xh}X_{t}\right)\times tanh$$

(3) $$f_{t}=\sigma \left(X_{t}W_{xf}+m_{t}W_{mf}\right)$$

(4) $$i_{t}=\sigma \left(X_{t}W_{xi}+m_{t}W_{mi}\right)$$

(5) $$o_{t}=\sigma \left(X_{t}W_{xo}+m_{t}W_{mo}\right)$$

(6) $$C_{t}=f_{t}⨂C_{t-1}+i_{t}⨂{\hat{h}}_{t}$$

(7) $$h_{t}=o_{t}⨂\tan h(C_{t})$$

In this example, ⨂ stands for element-by-element multiplication, $X_{t}$ represents the current input, $h_{t-1}$ remains for the previous hidden state, $C_{t-1}$ represents the previous unit state, σ stands for a sigmoid function, and tanh represents a tangent function.

### 2.3. Balancing Strategy

Classifiers were built from unbalanced datasets using the synthetic minority over-sampling technique (SMOTE) [33] methodology. SMOTE is an improved method based on the random oversampling algorithm [42], and primarily combines the analysis of minority class samples, the location of nearby samples, and the creation of artificially created new samples in accordance with the minority class samples. SMOTE first identifies the neighboring samples for all minority class samples using the k-nearest neighbors (KNN) algorithm, and then uses linear random interpolation for realizing sample synthesis. A random interpolation position is selected among the samples, and an equal number of interpolations are considered for each sample point. Such a balancing strategy for achieving data balance not only increases the sample size but also improves sample quality. Classifiers can learn more distinct features after processing with SMOTE, which significantly improves the performance of classifiers.

### 2.4. Feature Selection Strategy

We used three feature selection techniques, namely, analysis of variance (ANOVA) [43,44], light gradient boosting machine (LGBM) [6,45], and mutual information (MI) [46], for selecting the retrieved features. These techniques were employed in this study for determining the best feature space, and ranking the features based on their relevant ratings. The features with importance values larger than a crucial threshold (average feature importance value) were selected after sorting the features from the “largest” to the “smallest” based on the importance values.

#### 2.4.1. Analysis of Variance (ANOVA)

In this study, the features were sorted in order of importance using the ANOVA score. The mean difference between groups can be efficiently evaluated using ANOVA, which computes the ratio of variance within groups to the variance between groups for each feature [47]. The following formula was used for determining the ANOVA score:

(8) $$S\left(t\right)=\frac{S_{\theta }^{2}\left(t\right)}{S_{\omega }^{2}\left(t\right)}$$

where S(t) represents the score of the feature t, $S_{\theta }^{2}\left(t\right)$ stands for the variance between groups, and $S_{\omega }^{2}\left(t\right)$ is the variance within groups. The formulae used for calculating $S_{\theta }^{2}\left(t\right)$ and $S_{\omega }^{2}\left(t\right)$ are as follows:

(9) $$\begin{array}{c} S_{\theta }^{2}\left(t\right)=\frac{1}{K-1}\overset{K}{\underset{i=1}{\sum }}m_{i}{\left(\frac{\sum_{j=1}^{m_{i}}f_{t}\left(i,\;j\right)}{m_{i}}-\frac{\sum_{i=1}^{K}\sum_{j=1}^{m_{i}}f_{t}\left(i,\;j\right)}{\sum_{i=1}^{K}m_{i}}\right)}^{2} \end{array}$$

(10) $$S_{\omega }^{2}\left(t\right)=\frac{1}{N-K}\overset{K}{\underset{i=1}{\sum }}\overset{m_{i}}{\underset{j=1}{\sum }}{\left(f_{t}\left(i,\;j\right)-\frac{\sum_{j=1}^{m_{i}}f_{t}\left(i,\;j\right)}{m_{i}}\right)}^{2}$$

where K denotes the quantity of groups, N denotes the entire quantity of instances, and $f_{t}\left(i,\;j\right)$ denotes the value of the j-th sample in the i-th group of the feature t.

#### 2.4.2. Lighting Gradient Boosting Machine (LGBM)

LGBM [33] is a quick, dispersed, strong gradient boosting framework based on a decision tree technique that is employed in numerous ML applications, including classification and ranking. The gradient boosting decision tree (GBDT), which has the ability to learn the performances of learners, is continuously improving with several computational iterations. Here, we define $h_{c}\left(x\right)$ as an estimated function in Equation (11) and evaluate the loss function in Equation (12):

(11) $$h_{c}\left(x\right)=\arg\underset{h\in H}{\min}\sum L\left(y,F_{c-1}\left(x\right)+h\left(x\right)\right)$$

(12) $$r_{ti}=-\frac{\partial L(y,F_{t-1}\left(x_{i})\right)}{\partial F_{t-1}\left(x_{i}\right)}$$

where *c* means the current iteration, and $F_{c-n}\left(x\right)$ means the last *n* iterations’ model achievement. The following formula is used to select the most potential features in the current iteration, and the importance of each feature is obtained by ranking.

(13) $$F_{c+n}\left(x\right)=h_{2n}\left(x\right)+F_{c-n}\left(x\right)$$

#### 2.4.3. Mutual Information (MI)

MI has been widely used for feature selection since its development [48]. The advantage of MI in feature selection lies in its ability to equivalently define multidimensional variables and detect nonlinear relationships between variables. Owing to these advantages, the MI method can fully consider the joint correlation and redundancy of features during feature selection [49].

The entropy estimate for the peptide sequence S is provided in Equation (14):

(14) $$H\left(S\right)=-\underset{i\in \sum U}{\sum }P\left(\varepsilon_{i}\right)\log P\left(\varepsilon_{i}\right)$$

Using this entropy equation, the equation for the MI peptide sequence was deduced as:

(15) $$MI=\underset{i\in \sum U}{\sum }\underset{j\in \sum U}{\sum }P\left(\varepsilon_{i},\varepsilon_{j}\right)\log\frac{P\left(\varepsilon_{i},\varepsilon_{j}\right)}{P\left(\varepsilon_{i}\right)P\left(\varepsilon_{j}\right)}$$

where ∑U is the alphabet of amino acid residues and $P\left(\varepsilon_{i}\right)$ is the marginal probability of residue i.

### 2.5. Machine Learning Methods

Five widely used high-performance ML methods were used in this study, namely, KNN, linear regression (LR), support vector machine (SVM), random forest (RF), and LGBM [50].

KNN is one of the most straightforward machine learning algorithms that is better suited for automatic class categorization in studies with high sample sizes. Data are said to belong to a class if the minority of the K most comparable data in the feature space, or the feature space’s closest neighbors, also do. This approach only selects the class of the data to be based mainly on the classification of the data or the data nearest to it.

LR is categorized as a supervised learning method in ML. The concept of LR is that if data obey a certain distribution, then the parameters are estimated by maximum likelihood estimation. This method is actually a classification model and is often used for binary and multi-class classification problems. It is widely used owing to its simplicity, parallelizability, and strong interpretability.

SVM is applied for solving binary classification problems in bioinformatics. 

RF is a bagging-based technique that uses random feature selection during node splitting in addition to sampling at random.

LGBM is a gradient boosting framework that employs methods for learning from trees.

### 2.6. Evaluation Metrics and Methods

Five widely used measures were used for evaluating the performance of the models, and were calculated using Equations (16)–(20):

(16) $$\mathrm{ACC}=\frac{\mathrm{TP}+\mathrm{TN}}{\mathrm{FP}+\mathrm{FN}+\mathrm{TP}+\mathrm{TN}}$$

(17) $$\mathrm{MCC}=\frac{\mathrm{TP}\times \mathrm{TN}-\mathrm{FP}\times \mathrm{FN}}{\sqrt{\left(\mathrm{TP}+\mathrm{FP}\right)\left(\mathrm{TP}+\mathrm{FN}\right)\left(\mathrm{TN}+\mathrm{FP}\right)\left(\mathrm{TN}+\mathrm{FN}\right)}}$$

(18) $$\mathrm{Sn}=\frac{\mathrm{TP}}{\mathrm{TP}+\mathrm{FN}}$$

(19) $$\mathrm{Sp}=\frac{\mathrm{TN}}{\mathrm{TN}+\mathrm{FP}}$$

(20) $$\mathrm{BACC}=\frac{\mathrm{Sn}+\mathrm{Sp}}{2}$$

where TP denotes the amount of umami peptides successfully identified as umami, and TN denotes the quantity of non-peptides successfully identified as non-umami. FP denotes the amount of non-umami peptides falsely identified as umami, while FN denotes the quantity of umami peptides incorrectly identified as non-umami. The developed models were also contrasted with one another and with previously stated models based on the receiver operating characteristic curve (ROC). The area under the ROC curve (auROC) was also used for evaluating the predictive performance, where the values of auROC ranging between 0.5 and 1 stand for random and perfect models, respectively. The BACC approach is used for describing data imbalances, and the values of ACC and BACC are equal in a balanced sample.

K-fold cross-validation and independent testing methods are commonly used to evaluate ML models [51]. The raw data are separated into k-folds in K-fold cross-validation. The remaining K −1 subsets are utilized as training sets, while one subset is used for model validation. In the validation set, K models are evaluated separately, and the final values of the evaluation measures are averaged to obtain the cross-validated values. In this investigation, we employed the 10-fold (K = 10) cross-validation approach. The samples used in stand-alone testing were fresh for the trained model, and the test dataset used was completely different from the training set.

### 2.7. Cross-Entropy Loss

When performing a binary classification task, there are only positive and negative examples, and their probabilities add up to 1. Therefore, we simply need to predict a probability rather than a vector.

The loss function is defined simply as follows:

(21) $$Loss=-\left(y\cdot \log\left(\hat{y}\right)+\left(1-y\right)\cdot \log\left(1-\hat{y}\right)\right)$$

where *y* is the sample label, which takes the value of 1 if the sample is a positive case and 0 otherwise, and $\hat{y}$ is the probability that the model predicts that the sample is a positive case. In general, the lower the value of the cross-entropy loss function, the higher the classification effect [52,53,54,55].

## 3. Results and Discussion

### 3.1. Effect of SMOTE

**Figure 2:**
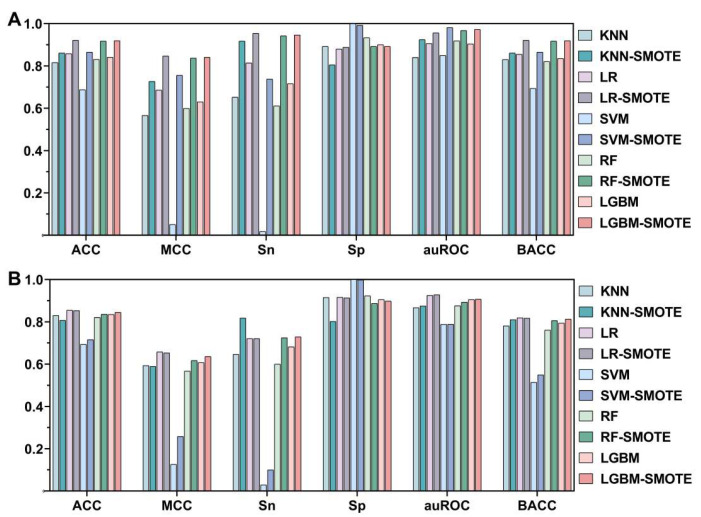
Results of 10-fold cross-validation (**A**) and independent testing (**B**) of the five ML models balanced with SMOTE and the five ML models balanced without SMOTE.As illustrated in Figure 2 and Supplementary Table S1, the features of models following optimization with SMOTE were clearly superior to the features of models developed without SMOTE optimization. Using the LR-based prediction model as an example, the LR-SMOTE model outperformed or equaled the LR model without SMOTE optimization in 66.7% of the metrics in 10-fold cross-validation and independent tests. Of the SVM-based models, the SVM-SMOTE model outperformed the SVM model developed without SMOTE optimization in 83.3% of the indicators.

We first extracted a 1900-dimensional feature vector using UniRep. The model was developed and initially trained using five different ML techniques, namely, KNN, LR, SVM, LGBM, and RF, for investigating the effect of SMOTE on the automatic identification of umami peptides. The outcomes of independent testing and 10-fold cross-validation of the five ML models optimized with SMOTE and five ML models optimized without SMOTE were obtained based on the aforementioned hypotheses, and are depicted in Figure 2 and Supplementary Table S1. The values in the tables and figures indicate model performance measures following the optimization of model parameters.

**Figure 3:**
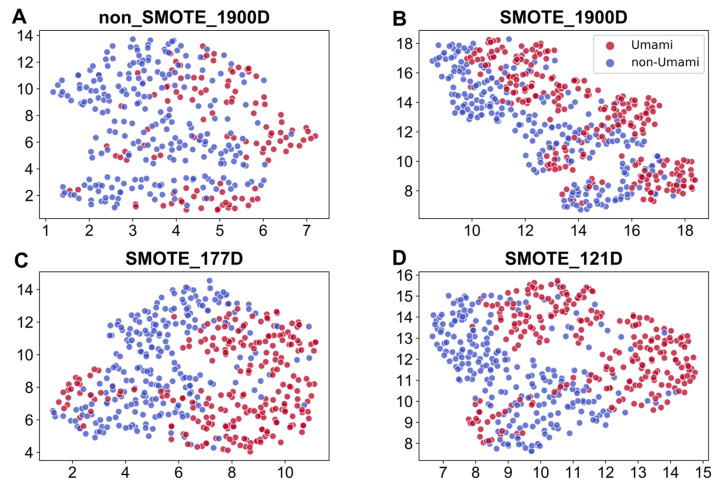
UMAP was used for visualizing the dimension-reduced features. (**A**) UniRep features without SMOTE balancing, (**B**) UniRep features following SMOTE balancing, (**C**) data of the top 177 features selected from the SMOTE-balanced UniRep feature set, and (**D**) data obtained using the top 121 features selected from the SMOTE-balanced UniRep feature set.

In some models, the Sp values were high while the Sn and other indicators were very poor owing to the bias of the unbalanced dataset bias towards the negative class, which negatively affected the recognition ability of the positive class. These findings also emphasize the value and significance of optimizing imbalanced datasets using SMOTE. However, it can be inferred from the UMAP display in Figure 3 that the improvement in the datasets using SMOTE improved the predictive ability of the models in identifying umami peptides.

### 3.2. Effects of Different ML Models

The results of Section 3.1 revealed that the SMOTE algorithm optimized the unbalanced data to a certain extent. The consequents of 10-fold cross-validation and independent tests of the models created using SMOTE-balanced features with the five ML algorithms are depicted in Table 1.

As depicted in Table 1, the recognition of umami peptides by the LR model outperformed that of the other ML models in 66.7% of the metrics. The consequents of 10-fold cross-validation revealed that the LR model, iUmami-DRLF, exceeded all other ML models in four metrics. The ACC and BACC of iUmami-DRLF were 0.22–6.97% superior to that of the other models, while the MCC and Sn increased by 0.71–16.51% and 0.85–29.27%, respectively. The results of the independent tests revealed that the LR model, iUmami-DRLF, outscored the other ML models in four metrics. The ACC, MCC, auROC, and BACC efficiency levels of iUmami-DRLF were superior to those of the other models by 0.95–19.13%, 2.67–153.10%, 2.32–17.62%, and 0.49–48.82%, respectively. Although the SVM model achieved the best indicators for the identification of umami peptides in certain aspects, the results of the independent tests revealed that the SVM model will show more unbalanced data (MCC = 0.258, Sn = 0.100, and BACC = 0.549). We, therefore, selected the LR model for developing the umami peptide predictor. Additionally, the results of the 10-fold cross-validation of the five models revealed that the values of ACC and BACC were equal, indicating that the dataset was balanced following optimization with SMOTE. The equal values of ACC and BACC have been indicated in blue in Table 1.

### 3.3. Effects of Different Feature Selection Methods

**Figure 4:**
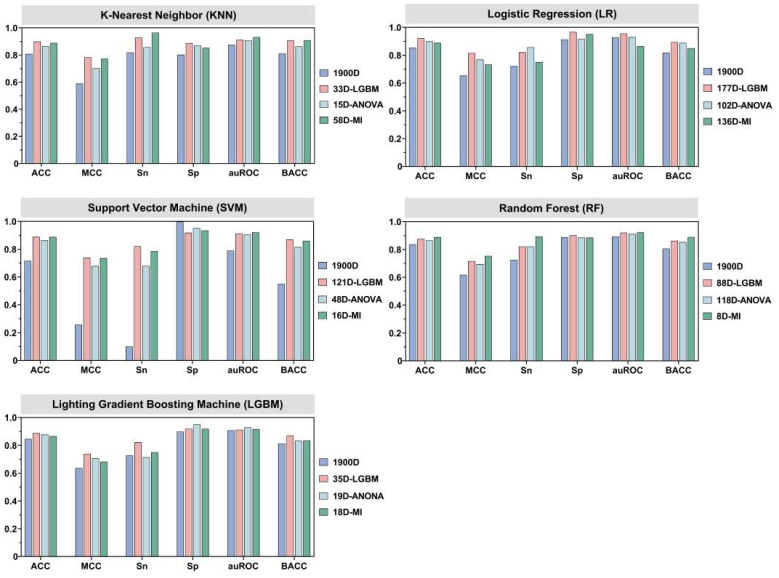
Comparison of the results of independent testing of the models with selected features and the models without selected features.

As described in Section 3.1, the balanced SMOTE-optimized data encoding method significantly outperformed the unprocessed data encoding approach in the tests. The feature vector that was recovered using UniRep had 1900 dimensions as opposed to the 353 dimensions of the sequence vector that was used in the training set. The use of high-dimensional feature vectors frequently leads to over-fitting or redundancy of feature information. In order to solve this issue, we used three feature selection methods, namely, ANOVA, LGBM, and MI, for selecting the high-dimensional feature vectors. An incremental feature strategy and a hyperparameter grid search approach were employed in this study, and the GridSearchCV module in the scikit-learn library was used for searching the hyperparameters for each model. Table 2 summarizes the outcomes of 10-fold cross-validation and independent testing of the five ML models developed based on the UniRep features selected using the three feature selection methods. The results of independent testing of the aforementioned models with selected features and the models without selected features are compared in Figure 4.

The outcomes of the independent testing are shown in Figure 4, which amply demonstrates that the chosen fusion feature sets outperformed the unselected fusion features. In the independent tests, the Sp of the 1900D models without feature selection was lower than all the models with feature selection, with the exception of the SVM-based model (5.00–8.75% higher). These results clearly demonstrated that the selection of feature descriptors effectively resolves information redundancy, and helps optimize the prediction performance of the umami peptide prediction model. Figure 4 and Table 2 clearly depict that of the three feature selection methods, and the overall performance of LGBM was superior to that of the other feature selection methods used for the identification of umami peptides. Considering the LR model as an example, the LGBM feature selection method outperformed the other methods (ANOVA and MI) in all six metrics in the 10-fold cross-validation studies. The performance of ACC, MCC, Sn, Sp, auROC, and BACC efficiency improved by 4.17–4.88%, 9.78–11.65%, 5.04–7.03%, 2.88–3.36%, 1.59–2.03%, and 4.17–4.88%, respectively, when the LGBM method was used. The LGBM feature selection method outperformed ANOVA and MI in the independent tests in five metrics. The performance of ACC, MCC, Sp, auROC, and BACC synergy improved by 2.45–3.72%, 6.12–11.19%, 1.68–5.34%, 2.80–10.65%, and 0.68–5.18%, respectively, when the LGBM method was used.

Based on the aforementioned results (Section 3.1, Section 3.2 and Section 3.3), we believe that the LR model developed based on the first 177 dimensions of UniRep using SMOTE-optimized data was superior in predicting umami peptides, and corroborates with the results of visual analysis discussed hereafter in Section 3.4. Based on the aforementioned analyses, the first 177D features of UniRep were selected for constructing the iUmami-DRLF predictor based on the LGBM model for subsequent studies.

### 3.4. Comparison with Existing Methods

In order to evaluate the efficacy and application of our technique in comparison to other predictors, we assessed and compared the predictive performance of iUmami-DRLF with that of other methods, including iUmami-SCM and UMPred-FRL. Table 3 compares the results of 10-fold cross-validation and independent testing of iUmami-DRLF with those of other existing methods.

Table 3 clearly demonstrates that iUmami-DRLF(SVM) outperformed the other classifiers in all metrics except Sp in the 10-fold cross-validation test. The ACC, MCC, Sn, auROC, and BACC of iUmami-DRLF(SVM) were superior to those of the other methods by 2.02–2.50%, 4.31–9.25%, 1.30–14.63%, 2.37–4.43%, and 2.02–4.77%, respectively. More significantly, the results of independent testing revealed that iUmami-DRLF(LR) performed better than the current predictors in every aspect. The ACC, MCC, Sp, auROC, and BACC were superior to those of the other methods by 3.76–6.51%, 10.86–20.00%, 4.51–15.05%, 4.04–6.47%, and 3.99–8.53%, respectively. 

Comparison of the two iUmami-DRLF predictors revealed that the results of the 10-fold validation of iUmami-DRLF(LR) were slightly worse than the results for the SVM model (ACC and BACC, MCC, Sn, Sp, and auROC were 2.02%, 4.31%, 1.30%, 2.79%, and 2.37% lower, respectively). However, the results of independent testing were superior for iUmami-DRLF(LR) (ACC, MCC, Sp, auROC, and BACC were 3.66%, 9.25%, 5.08%, 4.53%, and 2.75% higher, respectively). This revealed that the generalization ability of LR was stronger. The results of comparative analyses demonstrated the superiority of iUmami-DRLF in umami peptide prediction. The umami prediction ability of iUmami-DRLF was more reliable than the existing methods.

### 3.5. Feature Visualization

Feature visualization can intuitively convey feature information through images to clearly represent the dataset. UMAP is a popular uniform approximation projection algorithm for dimensionality reduction, and was used in this study for visual analyses of the features in the umami peptide dataset. The differences in feature representation are clearly highlighted in UMAP visualization. The results of dimensionality reduction for feature visualization with UMAP are depicted in Figure 3.

Figure 3 demonstrates that compared with the UniRep feature vector without SMOTE optimization (Figure 3A), the SMOTE-optimized 1900D UniRep feature vector (Figure 3B) was better at distinguishing umami peptides from non-umami peptides. Compared with the SMOTE-optimized UniRep features (Figure 3B), the top 177D features (Figure 3C) and top 121D features of UniRep (Figure 3D) were further optimized after feature selection.

### 3.6. Web Server Development

For other researchers to anticipate umami peptides, we created the user-friendly iUmami-DRLF web server, which is freely accessible online at https://www.aibiochem.net/servers/iUmami-DRLF/ (accessed on 1 April 2023). The web server is easy to use. The user only needs to enter the peptide sequence in the text box, click the run button, and wait for a few minutes, then the user can identify and judge whether the input peptide sequence is an umami peptide, and the result will be displayed on the web page. The output results include the input sequence, whether it is an umami peptide, and the confidence level. See the web server interface on the website or Supplementary Figures S1–S3. Additionally, please contact the corresponding authors if users need to predict a significant number of sequences.

### 3.7. Methods’ Robustness

To further verify the effectiveness and robustness of the model, we collected 91 wet-experiment verified umami peptide sequences reported in the latest literature [56,57,58,59,60,61,62,63,64,65,66,67,68,69,70]. These empirical umami peptide sequences constituted the dataset UMP-VERIFIED, which was then used to test state-of-the-art methods, including UMPred-FRL [11] and iUP-BERT [12] for comparison to iUmami-DRLF. Here, the accuracy of models under different prediction probability threshold conditions was adopted to make comparisons. The probability threshold T referred to the fact that, for a peptide sequence, if the probability threshold predicted by the machine learning model was greater than T, the model would determine that the sequence was an umami peptide; otherwise, it was a non-umami peptide.

**Figure 5:**
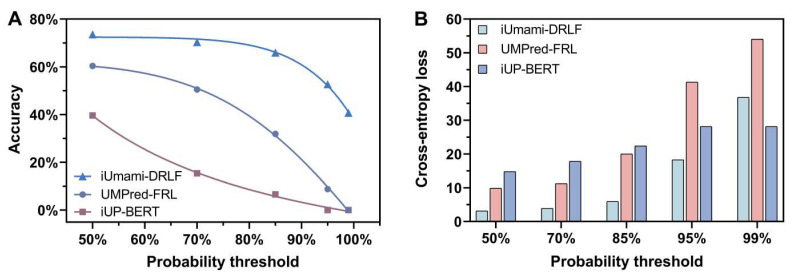
Under varying probability thresholds, the prediction results of iUmami-DRLF (this work), UMPred-FRL, and iUP-BERT are shown using the UMP-VERIFIED dataset. (**A**) is the relationship between prediction accuracy and probability threshold. (**B**) is the cross-entropy loss of the predicted outcome about the probability threshold. The smaller the cross-entropy loss, the better the robustness and accuracy of the model. Note that at the probability thresholds of 95% and 99%, the prediction accuracy of iUP-BERT and UMPred-FRL is 0, and their corresponding cross-entropy losses can be calculated, but they are not meaningful.

Figure 5A shows the relationship between the accuracy of the three models and the probability threshold. It can be seen from the figure that our model iUmami-DRLF has the best accuracy under any probability threshold. It was particularly noteworthy that the accuracy rate of iUP-BERT is 0 at 95% threshold probability, indicating that the model has failed. While the value of iUmami-DRLF is 52.7%, which is nearly six times the UMPred-FRL accuracy (8.8%), when the probability threshold was set to 99%, the prediction accuracy of iUP-BERT and UMPred-FRL is 0. It meant that both methods were invalid. In sharp contrast, iUmami-DRLF can still maintain the prediction accuracy of 40.7%, and the model still worked. These results proved that iUmami-DRLF is with better robustness and better model generalization performance than other methods.

The robustness and effectiveness of iUmami-DRLF come from the fact that it was an optimized model with minimum cross-entropy loss. For binary classification machine learning models, the closer the prediction output is to the real sample label, the smaller the cross-entropy loss is, resulting in better accuracy [71].

It could be proven by the data shown in Figure 5B. Figure 5B displays models’ cross-entropy loss under different probability thresholds. Obviously, iUmami-DRLF has the minimum cross entropy loss of the three models under the probability threshold of 50%, 70%, and 85%. At the probability threshold of 95%, the cross-entropy loss of iUmami-DRLF is significantly smaller than that of UMPred-FRL. For 95% and 99% probability thresholds, UMPred-FRL and iUP-BERT models have failed, and the calculated cross-entropy is meaningless. For example, the cross-entropy loss of iUP-BERT remains unchanged in probability thresholds of 95% and 99% of cases.

## 4. Conclusions and Future Work

In this research, we proposed a predictor, iUmami-DRLF, for the successful prediction of umami peptides solely based on sequence information. The imbalanced dataset was processed with SMOTE, and the latent umami peptide information was obtained using the UniRep deep representation learning feature embedding approach. Our predictor was strengthened by the use of three feature selection techniques, namely, LGBM, ANOVA, and MI, and the combination of five ML algorithms (KNN, LR, SVM, RF, and LGBM) for model development. Following testing and optimization, the top 177D features of UniRep were selected as the optimal feature set, and then integrated with the LR model for developing the final predictor. The results of 10-fold cross-validation and independent testing revealed that iUmami-DRLF markedly outperformed the existing methods in the independent tests. The latest umami peptide sequences verified by wet experiment were used to validate the method, and the results show that iUmami-DRLF could more reliably, robustly and accurately predict (independent tests: ACC = 0.921, MCC = 0.815, Sn = 0.821, Sp = 0.967, auROC = 0.956) umami peptides than the reported state-of-the-art methods. It hopes that the user-friendly webserver could be useful for researchers in the area. The following areas can still be improved, despite the fact that iUmami-DRLF has significantly increased the accuracy of umami peptide prediction: First, as our feature extraction model requires lots of computation, webservers without GPU configuration will take a long time to complete this task. Users can contact the corresponding authors if they need to predict a large number of sequences. Furthermore, using the most recent empirical data when training the model might produce better outcomes. Ultimately, using the method of model distillation can simplify the feature extraction model and lessen its computational complexity.

## Figures and Tables

**Table 1:** Results of 10-fold cross-validation and independent testing based on the five ML algorithms developed using SMOTE-balanced features.

Model	10-Fold Cross-Validation						Independent Test					
	ACC	MCC	Sn	Sp	auROC	BACC	ACC	MCC	Sn	Sp	auROC	BACC
LR ^c^	** 0.921 ** **^a^**	**0.847**	**0.954**	0.888	0.956	** 0.921 **	**0.853**	**0.653**	0.721	0.913	**0.928**	**0.817**
KNN ^c^	0.861 ^b^	0.727	0.917	0.805	0.924	0.861	0.807	0.589	**0.818**	0.802	0.875	0.810
SVM ^c^	0.865	0.756	0.738	**0.992**	**0.981**	0.865	0.716	0.258	0.100	**0.998**	0.789	0.549
RF^c^	0.917	0.837	0.942	0.892	0.967	0.917	0.836	0.617	0.725	0.887	0.893	0.806
LGBM ^c^	0.919	0.841	0.946	0.892	0.972	0.919	0.845	0.636	0.729	0.898	0.907	0.813

^a^ The best performance values are indicated in bold and underlined. ^b^ Blue indicates equal values of ACC and BACC. ^c^ LR: logistic regression; KNN: *k*-nearest neighbors; SVM: support vector machine; LGBM: light gradient boosting machine; RF: random forest.

**Table 2:** Results of 10-fold cross-validation and independent testing of the five ML models developed using UniRep features selected with the three feature selection methods (LGBM, ANOVA, and MI).

Model	Feature Selection Method	Dim	10-Fold Cross-Validation						Independent Test					
			ACC	MCC	Sn	Sp	auROC	BACC	ACC	MCC	Sn	Sp	auROC	BACC
LR ^c^	LGBM ^d^	177	0.925 ^b^	0.853	0.959	0.892	0.957	0.925	**0.921 ** ^a^	**0.815**	0.821	**0.967**	**0.956**	0.894
	ANOVA ^d^	102	0.882	0.764	0.896	0.867	0.938	0.882	0.899	0.768	0.857	0.918	0.930	0.888
	MI ^d^	136	0.888	0.777	0.913	0.863	0.942	0.888	0.888	0.733	0.750	0.951	0.864	0.850
KNN ^c^	LGBM ^d^	33	0.892	0.788	0.938	0.846	0.955	0.892	0.899	0.782	0.929	0.885	0.911	0.907
	ANOVA ^d^	15	0.873	0.748	0.896	0.851	0.934	0.873	0.865	0.703	0.857	0.869	0.907	0.863
	MI ^d^	58	0.888	0.783	0.954	0.822	0.927	0.888	0.888	0.773	**0.964**	0.852	0.931	**0.908**
SVM ^c^	LGBM ^d^	121	** 0.944 **	**0.889**	**0.971**	**0.917**	0.980	** 0.944 **	0.888	0.739	0.821	0.918	0.913	0.870
	ANOVA ^d^	48	0.925	0.854	0.967	0.884	0.977	0.925	0.865	0.678	0.679	0.951	0.906	0.815
	MI^d^	16	0.919	0.841	0.959	0.880	0.968	0.919	0.888	0.735	0.786	0.934	0.921	0.860
RF ^c^	LGBM ^d^	88	0.915	0.830	0.934	0.896	0.975	0.915	0.876	0.716	0.821	0.902	0.920	0.862
	ANOVA ^d^	118	0.898	0.797	0.913	0.884	0.961	0.898	0.865	0.694	0.821	0.885	0.911	0.853
	MI ^d^	8	0.902	0.806	0.921	0.884	0.952	0.902	0.888	0.753	0.893	0.885	0.923	0.889
LGBM ^c^	LGBM ^d^	35	0.938	0.877	**0.971**	0.905	**0.988**	0.938	0.888	0.739	0.821	0.918	0.912	0.870
	ANOVA ^d^	19	0.902	0.807	0.942	0.863	0.945	0.902	0.876	0.706	0.714	0.951	0.929	0.833
	MI ^d^	18	0.888	0.777	0.917	0.859	0.953	0.888	0.865	0.682	0.750	0.918	0.916	0.834

^a^ The best performance values are indicated in bold and underlined. ^b^ Blue indicates equal values of ACC and BACC. ^c^ LR: logistic regression; KNN: *k*-nearest neighbors; SVM: support vector machine; LGBM: light gradient boosting machine; RF: random forest. ^d^ LGBM: light gradient boosting machine; ANOVA: analysis of variance; MI: mutual information.

**Table 3:** Results of 10-fold cross-validation and independent testing of iUmami-DRLF and other existing methods.

Classifier	10-Fold Cross-Validation						Independent Test					
	ACC	MCC	Sn	Sp	auROC	BACC	ACC	MCC	Sn	Sp	auROC	BACC
iUmami-DRLF(LR)	0.925 ^b^	0.853	0.959	0.892	0.957	0.925	**0.921 ** ^a^	**0.815**	0.821	**0.967**	**0.956**	0.894
iUmami-DRLF(SVM)	** 0.944 **	**0.889**	**0.971**	0.917	**0.980**	** 0.944 **	0.888	0.739	0.821	0.918	0.913	0.870
iUP-BERT	0.940	0.881	0.963	0.917	0.971	0.940	0.899	0.774	**0.893**	0.902	0.933	**0.897**
UMPred-FRL	0.921	0.814	0.847	**0.955**	0.938	0.901	0.888	0.735	0.786	0.934	0.919	0.860
iUmami-SCM	0.935	0.864	0.947	0.930	0.945	0.939	0.865	0.679	0.714	0.934	0.898	0.824

^a^ The best performance values are indicated in bold and underlined. ^b^ Blue indicates equal values of ACC and BACC.

## Data Availability

The data used to support the findings of this study can be made available by the corresponding author upon request.

## Supplementary Materials

The following supporting information can be downloaded at: https://www.mdpi.com/article/10.3390/foods12071498/s1, Figures S1–S3: iUmami-DRLF web server interface; Table S1: Results of 10-fold cross-validation and independent testing about SMOTE; Table S2: Prediction-probability results of the three models and 91 wet-experiment validated umami peptide sequences [5,6,11,56,59,60,62,63,64,65,66,67,68,69,70].
