# Supplementary material for: A Machine Learning Method to Identify Umami Peptide Sequences by Using Multiplicative LSTM Embedded Features
Source: Foods. 2023 Apr 2;12(7):1498. doi: 10.3390/foods12071498 (PMC10094688; doi:10.3390/foods12071498)
Supplement: Supplementary file 1 [file foods-12-01498-s001.zip › foods-2277139-supplementary.pdf]

Supplementary table legends:

Supplementary Table S1. Results of 10-fold cross-validation and independent testing of the five machine learning (ML) models balanced with synthetic minority over-sampling technique (SMOTE) and the five ML models balanced without SMOTE.

Model	10-fold cross-validation						Independent test					
	ACC	MCC	Sn	Sp	auROC	BACC	ACC	MCC	Sn	Sp	auROC	BACC
KNN[c]	0.816	0.566	0.652	0.892	0.840	0.830	0.830	0.593	0.646	0.915	0.867	0.781
KNN-SMOTE[c]	0.861[b]	0.727	0.917	0.805	0.924	0.861	0.807	0.589	**0.818**[a]	0.802	0.875	0.810
LR[c]	0.858	0.686	0.814	0.880	0.906	0.855	**0.855**	**0.658**	0.721	0.916	0.925	**0.819**
LR-SMOTE[c]	**0.921**	**0.847**	**0.954**	0.888	0.956	**0.921**	0.853	0.653	0.721	0.913	**0.928**	0.817
SVM[c]	0.688	0.051	0.018	**1.000**	0.850	0.694	0.694	0.126	0.029	**1.000**	0.788	0.514
SVM-SMOTE[c]	0.865	0.756	0.738	0.992	**0.981**	0.865	0.716	0.258	0.100	0.998	0.789	0.549
RF[c]	0.831	0.599	0.611	0.933	0.918	0.821	0.821	0.567	0.600	0.923	0.876	0.761
RF-SMOTE[c]	0.917	0.837	0.942	0.892	0.967	0.917	0.836	0.617	0.725	0.887	0.893	0.806
LGBM[c]	0.841	0.630	0.716	0.900	0.904	0.835	0.835	0.607	0.682	0.905	0.905	0.794
LGBM-SMOTE[c]	0.919	0.841	0.946	0.892	0.972	0.919	0.845	0.636	0.729	0.898	0.907	0.813

[a]The best performance values are indicated in bold and underlined.

[b]Blue indicates equal values of ACC and BACC.

[c]LR: logistic regression; KNN: *k*-nearest neighbors; SVM: support vector machine; LGBM: light gradient boosting machine; RF: random forest. SMOTE: synthetic minority over-sampling technique.

Supplementary Table S2. Prediction-probability results of the three models and details of 91 wet-experiment validated umami peptide sequences collected from the latest research.

ID	Seq	REF	● Umami	Prediction-probability		
				iUmami-DRLF	iUP-BERT	UMPred-FRL
1	EFLKEQF	[1]	● YES	12.30%	48.30%	45.80%
2	KALSEELDN	[1]	● YES	98.88%	86.59%	22.60%
3	AAEAKLELLE	[1]	● YES	99.56%	75.09%	86.50%
4	KETLEQEKSG	[1]	● YES	99.18%	56.81%	87.30%
5	TWKE	[1]	● YES	99.42%	39.79%	10.20%
6	DLRAD	[1]	● YES	99.63%	76.87%	28.40%
7	DDLDR	[1]	● YES	93.33%	38.01%	79.30%
8	KSLEEA	[1]	● YES	99.70%	72.43%	93.50%
9	MQAMKLEK	[1]	● YES	89.05%	60.34%	88.60%
10	DQLEKQVK	[1]	● YES	99.51%	43.44%	76.80%
11	AKALKEEDL	[1]	● YES	99.91%	77.03%	10.20%
12	TGFLPEEYMK	[1]	● YES	41.84%	24.68%	42.10%
13	AMKLEKENAL	[1]	● YES	96.87%	61.80%	57.30%
14	VADLMR	[2]	● YES	14.81%	39.60%	58.80%
15	STELFK	[2]	● YES	86.09%	57.79%	73.20%
16	DALKKK	[2]	● YES	97.78%	25.19%	70.10%
17	FVGLQER	[2]	● YES	34.05%	51.04%	25.80%
18	VVLNPVARVE	[2]	● YES	94.30%	48.22%	33.00%
19	AGFMPLP	[3]	● YES	0.09%	11.40%	28.20%
20	APYSGY	[3]	● YES	27.94%	15.10%	2.50%
21	PPMF	[3]	● YES	0.04%	1.16%	5.40%
22	SLSSLMK	[3]	● YES	4.20%	28.35%	67.90%
23	VAMNPVDHPH	[3]	● YES	30.82%	11.49%	88.60%
24	DK	[4]	● YES	99.96%	85.56%	96.90%
25	EEK	[4]	● YES	94.86%	56.69%	95.60%
26	EDQK	[4]	● YES	97.17%	94.10%	98.50%
27	SEGR	[4]	● YES	85.65%	89.48%	94.20%
28	QDSIGS	[4]	● YES	61.64%	81.11%	34.60%
29	RD	[5]	● YES	98.99%	39.30%	98.80%
30	DGV	[5]	● YES	99.84%	35.04%	77.80%
31	FSGLDGAK	[6]	● YES	3.23%	58.67%	11.80%
32	FAGDDAPR	[6]	● YES	14.50%	43.70%	29.40%
33	FSGLDGSK	[6]	● YES	1.37%	53.35%	9.70%
34	DGF	[7]	● YES	95.37%	60.34%	18.30%
35	KCGQ	[7]	● YES	100.00%	30.61%	36.70%
36	HHYE	[7]	● YES	0.00%	22.12%	90.70%

37	INKPGL	[8]	● YES	1.03%	7.54%	11.40%
38	SDSCI	[8]	● YES	100.00%	19.77%	76.40%
39	GPDPER	[8]	● YES	90.69%	82.00%	54.60%
40	TGC	[9]	● YES	100.00%	45.52%	79.00%
41	GLE	[9]	● YES	2.34%	28.90%	35.50%
42	VEAL	[9]	● YES	98.90%	20.87%	67.20%
43	GGE	[9]	● YES	99.32%	52.08%	5.00%
44	EVC	[9]	● YES	100.00%	23.78%	93.90%
45	DAE	[9]	● YES	100.00%	88.06%	88.20%
46	EPLCNQ	[10]	● YES	100.00%	43.83%	46.90%
47	SGCVNEL	[10]	● YES	100.00%	82.26%	91.20%
48	PHEMQ	[10]	● YES	91.12%	59.55%	78.80%
49	SEPSHF	[10]	● YES	95.38%	66.58%	82.00%
50	ESCAPQL	[10]	● YES	100.00%	81.36%	68.80%
51	MTNLLEDLSFR	[11]	● YES	4.04%	30.83%	77.50%
52	GFGDSCTPGKNER	[11]	● YES	100.00%	31.15%	73.80%
53	YADSNIQINGTDR	[11]	● YES	64.55%	51.39%	40.90%
54	AREALQELGEQAK	[11]	● YES	99.82%	29.70%	29.60%
55	IQQDDCK	[11]	● YES	100.00%	43.34%	98.70%
56	QAEADAR	[11]	● YES	100.00%	66.68%	92.10%
57	HLQLAIR	[12]	● YES	0.10%	15.22%	35.50%
58	DPLRGGYY	[12]	● YES	8.80%	17.46%	4.60%
59	AGLQFPVGR	[12]	● YES	2.24%	12.83%	11.10%
60	LLLPGELAK	[12]	● YES	19.02%	7.31%	89.80%
61	AGFAGDDAPR	[12]	● YES	5.33%	34.18%	46.50%
62	GYSFTTTAER	[12]	● YES	99.89%	50.00%	32.00%
63	DAGVIAGLNVLR	[12]	● YES	2.91%	20.93%	91.60%
64	HEAL	[13]	● YES	88.70%	37.71%	73.20%
65	LREY	[13]	● YES	98.93%	31.87%	94.50%
66	LAKVH	[13]	● YES	99.47%	30.71%	75.00%
67	FQKVVA	[13]	● YES	64.51%	40.28%	90.00%
68	HVKELE	[13]	● YES	94.26%	57.35%	74.30%
69	AEVKKAP	[13]	● YES	92.08%	66.53%	79.00%
70	KALSEEL	[13]	● YES	99.87%	93.97%	80.40%
71	KKMFETES	[13]	● YES	98.09%	62.93%	88.90%
72	EAVEKPQS	[13]	● YES	73.60%	50.00%	96.90%
73	DAPYDYK	[14]	● YES	41.19%	33.97%	50.00%
74	LLDAFFFDNK	[14]	● YES	10.39%	21.24%	4.90%
75	MAASGDVGK	[14]	● YES	74.19%	57.44%	32.30%
76	NALKSVECYDAR	[14]	● YES	100.00%	42.99%	88.90%
77	AKLTSLEEECQR	[14]	● YES	100.00%	30.53%	95.80%
78	YLASCLSSVKEEK	[14]	● YES	100.00%	22.59%	35.50%

79	MEREQEESTMR	[14]	● YES	79.35%	28.19%	96.20%
80	SGVVAAVNDAAKDFHG	[14]	● YES	99.17%	36.69%	48.20%
81	VLSLNSGTEAVEAAIK	[14]	● YES	97.84%	52.04%	44.20%
82	DDF	[15]	● YES	99.82%	23.34%	94.60%
83	ADC	[15]	● YES	100.00%	23.69%	94.10%
84	DNW	[15]	● YES	99.74%	19.28%	13.20%
85	QDF	[15]	● YES	99.70%	63.34%	50.00%
86	IGDM	[15]	● YES	91.50%	40.07%	92.40%
87	DCIY	[15]	● YES	100.00%	25.78%	88.30%
88	AGCD	[15]	● YES	99.98%	38.16%	74.50%
89	QDTW	[15]	● YES	99.98%	22.14%	6.70%
90	SGDAW	[15]	● YES	99.22%	27.35%	20.80%
91	NDDGW	[15]	● YES	79.23%	22.24%	52.90%

References

1. Zhang, T.; Hua, Y.; Zhou, C.; Xiong, Y.; Pan, D.; Liu, Z.; Dang, Y., Umami peptides screened based on peptidomics and virtual screening from Ruditapes philippinarum and Mactra veneriformis clams. *Food Chemistry* **2022,** 394, 133504.
2. Shiyan, R.; Liping, S.; Xiaodong, S.; Jinlun, H.; Yongliang, Z., Novel umami peptides from tilapia lower jaw and molecular docking to the taste receptor T1R1/T1R3. *Food Chemistry* **2021,** 362, 130249.
3. Gao, B.; Hu, X.; Xue, H.; Li, R.; Liu, H.; Han, T.; Ruan, D.; Tu, Y.; Zhao, Y., Isolation and screening of umami peptides from preserved egg yolk by nano-HPLC-MS/MS and molecular docking. *Food Chemistry* **2022,** 377, 131996.
4. Yu, Z.; Kang, L.; Zhao, W.; Wu, S.; Ding, L.; Zheng, F.; Liu, J.; Li, J., Identification of novel umami peptides from myosin via homology modeling and molecular docking. *Food Chemistry* **2021,** 344, 128728.
5. Cao, C.; Wang, J.; Kwok, D.; Cui, F.; Zhang, Z.; Zhao, D.; Li, M. J.; Zou, Q., webTWAS: a resource for disease candidate susceptibility genes identified by transcriptome-wide association study. *Nucleic Acids Research* **2022,** 50, (D1), D1123-d1130.
6. Jiang, J.; Lin, X.; Jiang, Y.; Jiang, L.; Lv, Z., Identify Bitter Peptides by Using Deep Representation Learning Features. *International Journal of Molecular Sciences* **2022,** 23, (14), 7877.
7. Song, S.; Zhuang, J.; Ma, C.; Feng, T.; Yao, L.; Ho, C.-T.; Sun, M., Identification of novel umami peptides from Boletus edulis and its mechanism via sensory analysis and molecular simulation approaches. *Food Chemistry* **2023,** 398, 133835.
8. Zhu, W.; He, W.; Wang, F.; Bu, Y.; Li, X.; Li, J., Prediction, molecular docking and identification of novel umami hexapeptides derived from Atlantic cod (Gadus morhua). *International Journal of Food Science & Technology* **2021,** 56, (1), 402-412.
9. Zhu, X.; Sun-Waterhouse, D.; Chen, J.; Cui, C.; Wang, W., Comparative study on the novel umami-active peptides of the whole soybeans and the defatted soybeans fermented soy sauce. *Journal of the Science of Food and Agriculture* **2021,** 101, (1), 158-166.
10. Chen, W.; Li, W.; Wu, D.; Zhang, Z.; Chen, H.; Zhang, J.; Wang, C.; Wu, T.; Yang, Y., Characterization of novel umami-active peptides from Stropharia rugoso-annulata mushroom and in silico study on action mechanism. *Journal of Food Composition and Analysis* **2022,** 110, 104530.
11. Charoenkwan, P.; Nantasenamat, C.; Hasan, M. M.; Moni, M. A.; Manavalan, B.; Shoombuatong, W., UMPred-FRL: A New Approach for Accurate Prediction of Umami Peptides Using Feature Representation Learning. *International Journal of Molecular Sciences* **2021,** 22, (23), 13124.
12. Liu, Z.; Zhu, Y.; Wang, W.; Zhou, X.; Chen, G.; Liu, Y., Seven novel umami peptides from Takifugu rubripes and their taste characteristics. *Food Chemistry* **2020,** 330, 127204.

13. Liu, Q.; Gao, X.; Pan, D.; Liu, Z.; Xiao, C.; Du, L.; Cai, Z.; Lu, W.; Dang, Y.; Zou, Y., Rapid screening based on machine learning and molecular docking of umami peptides from porcine bone. *Journal of the Science of Food and Agriculture* **2022**.

14. Zhu, W.; Luan, H.; Bu, Y.; Li, X.; Li, J.; Zhang, Y., Identification, taste characterization and molecular docking study of novel umami peptides from the Chinese anchovy sauce. *Journal of the Science of Food and Agriculture* **2021,** 101, (8), 3140-3155.

15. Wang, Y.; Luan, J.; Tang, X.; Zhu, W.; Xu, Y.; Bu, Y.; Li, J.; Cui, F.; Li, X., Identification of umami peptides based on virtual screening and molecular docking from Atlantic cod (Gadus morhua). *Food & Function* **2023,** 14, (3), 1510-1519.

Supplementary figures legends:

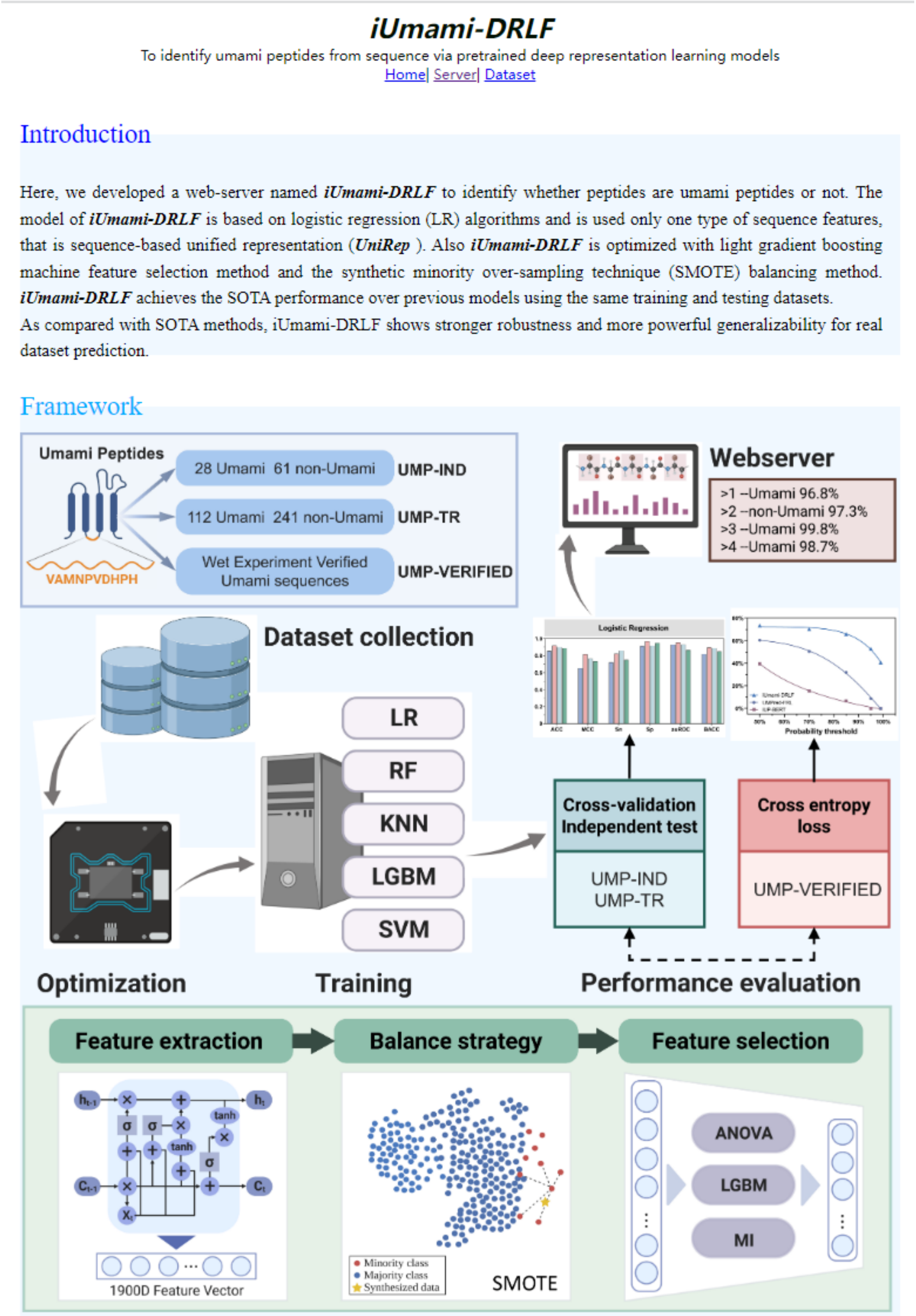

Supplementary Figure S1. Screenshots of the iUmami-DRLF webserver. Users can access the webserver by entering https://www.aibiochem.net/servers/iUmami-DRLF/, which would bring us to the webpage as shown in Supplemental Figure 1.

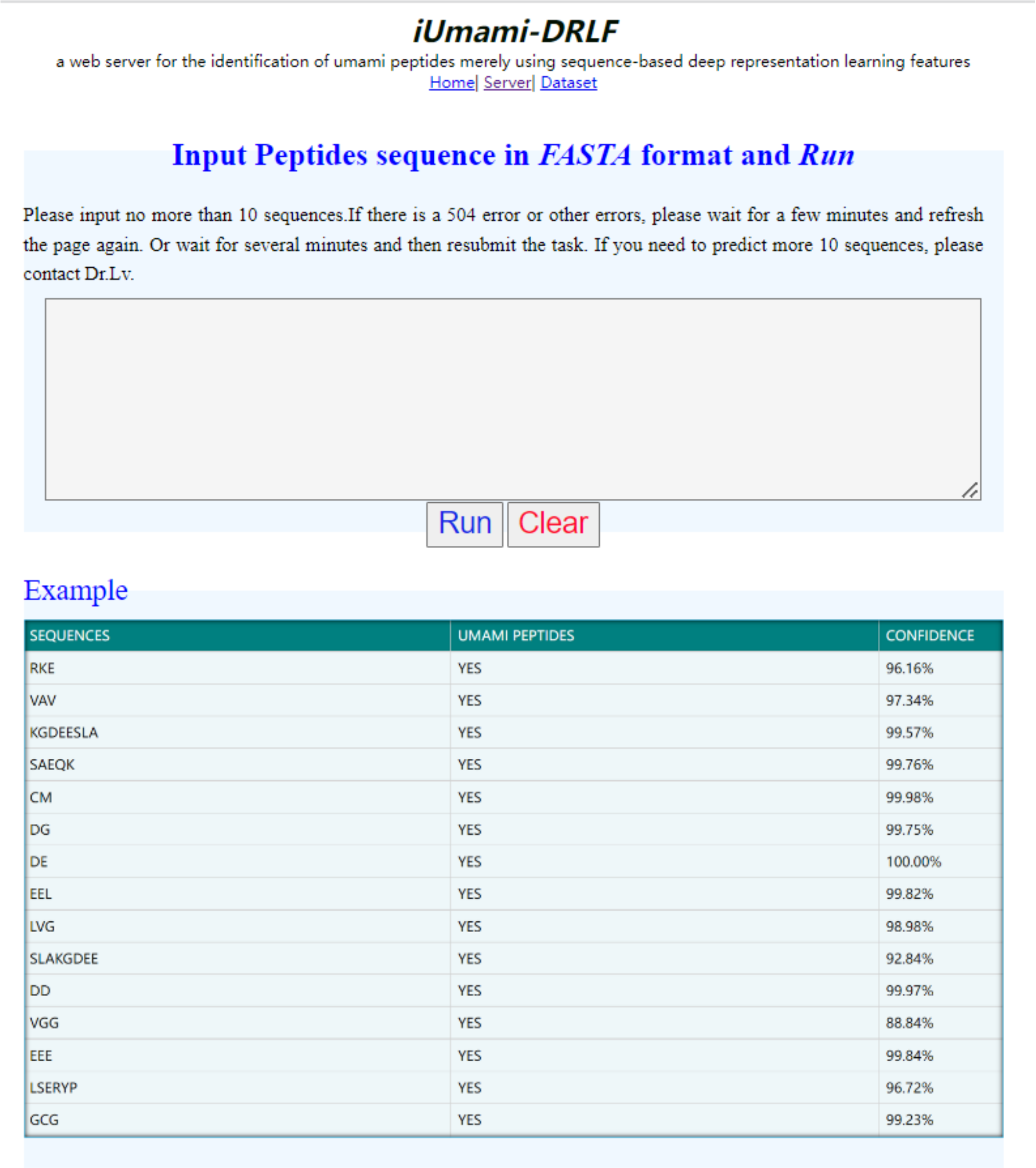

SEQUENCES	UMAMI PEPTIDES	CONFIDENCE
RKE	YES	96.16%
VAV	YES	97.34%
KGDEESLA	YES	99.57%
SAEQK	YES	99.76%
CM	YES	99.98%
DG	YES	99.75%
DE	YES	100.00%
EEL	YES	99.82%
LVG	YES	98.98%
SLAKGDEE	YES	92.84%
DD	YES	99.97%
VGG	YES	88.84%
EEE	YES	99.84%
LSERYP	YES	96.72%
GCG	YES	99.23%

Supplementary Figure S2. Screenshots of the iUmami-DRLF webserver. Users can begin the prediction process by entering the query sequence into the text box and clicking the "Run" button; this step typically takes a few seconds for the server to process the task.

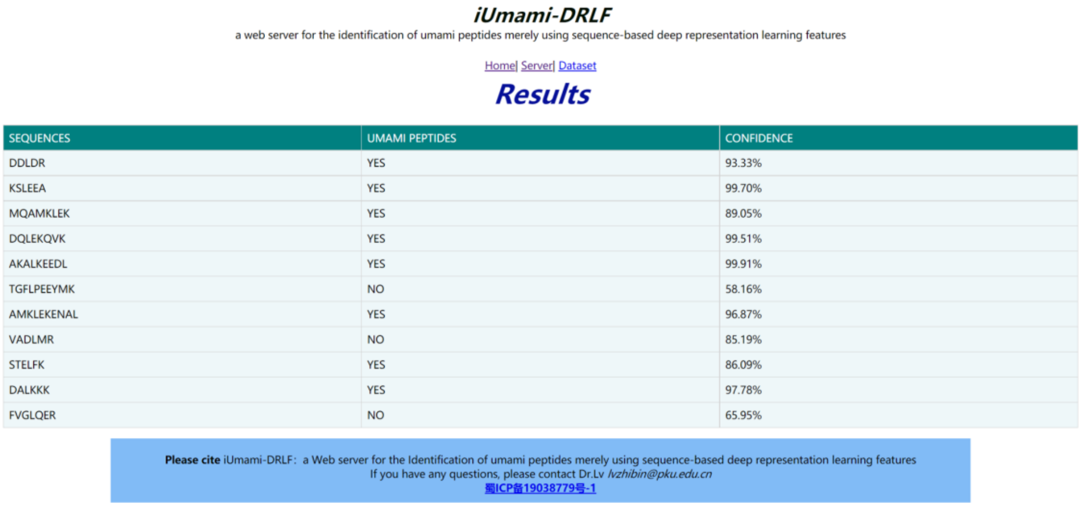

Supplementary Figure S3. Screenshots of the iUmami-DRLF webserver. After the prediction process is completed, the results are displayed on the webserver.
